# InterspeciesForms the hybridization of architectural, biological and robotic agencies

**DOI:** 10.1007/s44223-023-00025-0

**Published:** 2023-03-20

**Authors:** Natalie Alima

**Affiliations:** School of Architecture and Urban Design, RMIT University, Melbourne, Australia

**Keywords:** Mycelium, Robotic feedback, Hybridization, Computational design

## Abstract

Situated in the field of architectural biodesign, InterspeciesForms explores a closer relationship between the fungus Pleurotus ostreatus and the designer in the creation of form. The intention of hybridizing mycelia’s agency of growth with architectural design aesthetic, is to generate novel, non- indexical crossbred designed outcomes. The purpose of this research to advance architecture's existing relationship with the biological and evolve preconceived notions of form. In order to establish a direct dialogue between architectural and mycelia agencies, robotic feedback systems are implemented to extract data from the physical realm and feed it into the digital. Initiating this cyclic feedback system, mycelia growth is scanned in order to computationally visualize its entangled network and agency of growth. Utilizing mycelia’s physical data as impute, the architect then embeds design intention into this process through customized algorithms based on the logic of stigmergy. In order to bring this cross-bred computational outcome back into the physical realm, form is 3D printed with a customized mixture of mycelium and agricultural waste. Once the geometry has been extruded, the robot patiently waits for the mycelia to grow and react to the organic 3D- printed compound. The architect then responds with a countermove, by scanning this new growth and continuing the cyclic feedback system between nature-machine and the architect. This procedure demonstrates form emerging in real time according to the co-creational design process and dynamic dialogue between architectural and mycelia agencies.

## Introduction

Mycelium are threadlike fibrous root systems made up of hyphae, that form the vegetative part of a fungus (Lim, 2022). Known as the hackers of the wood wide web (Simard et al. 1997) mycelia forms complex symbiotic relationships with other species that inhabit our earth, as Michael Lim states “Fungi redefine resourcefulness, collaboration, resilience and symbiosis” (Lim and Shu, 2022, p. 14). When wondering around the forest to connect with other species or searching for food, fungi form elaborate and entangled networks by spreading their hyphal tips. This living labyrinth results in the aesthetic formation of an intricate web. Darwin illustrated that root tips act like a brain as they link perception and action, and determine the trajectory of growth (Darwin, 1859). Sheldrake (2021) links this behaviour to fungal hyphae, as data is streamed through the organism’s tips, determining the speed and direction of growth. The Latin root of the word intelligence means “to choose between” (Sheldrake, 2021, p. 73), suggesting a form of intelligence expressed through the mycelia’s ‘decision gates’. Due the organisms ability to determine the most effective direction of growth, communicate with its surrounding ecosystem and connect with other species, fungi are indeed an intelligent species with a unique aesthetic, that must not be ignored. In drawing on these concepts, I refer to the organism’s ability to search for, tangle and digest its surroundings as ‘mycelia agency of growth’. It is this specific behavioural characteristic that is the focus of this research, with which I as the architect set out to co-create and hybridize with.

## Mycelium and architecture

Over the years, a vast amount of interest and research has developed, testing fungi’s compressive strength, acoustic absorption, fire safety properties and application to architecture. Due to the organisms ability to up-cycle materials and biodegrade them, fungal mycelium is often considered a sustainable alternative to synthetic materials (Jones et al. 2018). Mycelium-derived materials have several key advantages over traditional synthetic materials, including their: low cost, density, and energy consumption; their ability to biodegrade; and their overall low environmental impact and carbon footprint (Haneef et al. 2017). David Benjamin for example has successfully converted mycelium into known architectural applications such as bricks. Displayed in the courtyard of MoMA's PS1 space in New York Hy-Fi Tower (2014), Benjamin demonstrates the fabrication of organic, biodegradable bricks made of farm waste and a culture of fungus that was grown to fit a brick-shaped mold. In a similar approach, Phillipe Block’s MycoTree installation (2017) is made of load-bearing mycelium components that replaces know architectural applications such as columns. Comprised of mycelium-infused waste that is ordinarily weak in tension, Block’s research team managed to hack into the mycelia’s structural capabilities through form (Heisel et al. 2017). Advancing this fabrication method further, Pulp Faction by Ana Goidea, David Andreen and Dimitrios Floudas, demonstrates the robotic extrusion of mycelium infused with agricultural waste. This innovative research utilizes local organic waste in order to replace the existing petroleum based plastics that are commonly used for 3D printing such as Polyethylene terephthalate glycol (PETG). To convert the living organism into a material that is suitable for the architectural field, the following process occurred within all three precedent projects listed above:

The mycelium medium was initially grown on agricultural waste which provided nutrients for the organism to flourish. In the case of Phillipe Block’s MycoTree and David Benjamin’s Hy-Fi Tower, the mixture was then infused into predetermined digitally fabricated moulds in order for the organism to adopt the generated form. Following the growth period, the composite mixture is removed from the mould and either hot-pressed or oven dried which dehydrates the material and neutralizes the fungus (Jones et al. 2020). By applying extreme heat to the fungus, this process converts the once living organism into a pre-formulated static building material by ensuring that it does not grow past the required shape (Holt et al. 2012). In the case of ‘Pulp Faction’ mycelia is grown on agricultural waste and robotically extruded, illuminating the need for a digitally fabricated mould. In a similar process to MycoTree and Hy-Fi Tower, once the living organism has been 3D printed, extreme heat is applied in order to ensure that the organism is no longer able to grow, adapt, or respond past its required geometric shape.

InterspeciesForms posits that by making nature inert or converting it into static materials, ‘appropriate’ for the building industry, a crucial, biological- driven design process is being ignored. By converting mycelium into static non responsive materials, this research posits that fungi’s agency and true contribution to architecture has yet to be explored. This suggest that designing predictable and replicable forms, that are reflective of an architect’s aesthetics, are impossible to achieve without compromising the inherent flourishing^1^ needs of the fungus. Consequently, this establishes a predominantly instrumental relationship with nature, where nature was conceived as the supplier of materials and context, but rarely invited to actively participate in the design, and even far less so to have agency and autonomy in the creation of forms. This existing process of infusing biological materials into static, predetermined forms must be inverted as a crucial, material-driven design process is missing. Rather than ignoring natures agency, this research seeks to examine the potential contributions of the fungus’s aesthetics to architectural design. From this perspective, the mycelia’s properties of growth are essential for the creation of novel forms and advancing the field of biodesign. InterspeciesForms therefore explores the possibilities of fungi that transcend its application of a sustainable material.

## Hybridization

In adopting a bio egalitarian view related to co-designing with the more-than-human world, this research put forward the following aims: to contribute to developing our understanding regarding architect–non-human organism relationships and their affordances to architectural design, and to develop new methodologies that facilitate the agency of the non-human organisms, while contributing to design innovation. The findings of this research (described below) provide the empirical grounding for reconceptualising and redefining the notion of hybridization within architectural design. By embracing nature’s intelligence and behavioural characteristics, new biologically driven design processes may emerge. The importance of generating hybridisation between architectural design intention and biological agency enables a dynamic shift from predicable outcomes, into highly volatile and dynamic form finding mythologies.

Thus far, architects have tended to define hybridization through a human centric perspective, where nature often has minimal agency or autonomy within the generation of form. Specifically within the field of biodesign, often the mere presence of non-human organisms in the outcome of form is considered hybridization. This narrow perception suggests that the mixing of living and non-living materials qualifies the material to be considered a hybrid, regardless of any considerations to the qualitative properties of the emerging design (Alima, 2022). In this sense, natures potential contribution to the aesthetic of the form is limited and bounded by the architect’s imagination. Based on the empirical findings of this research, InterspeciesForms posits the following definition for true hybridization:

*Hybridisation in forms co-created by human and non-human organisms must meet the criterion of presenting a non-indexical formation in relation to essential properties of the form, such as aesthetics and scale* (Alima, 2022).

The potentials of this ‘partnership’ with non-human organism’s, is to not only expand the imagination of the designer, but to generate novel forms which otherwise would not have been generated if designing individually. Michael Lim further explains this notion that organism’s should not be understood in isolation when stating “Fungi teach us that we are all interdependent. When we finally surrender our separateness, we realize that we are not outside of nature, but with it” (Lim and Shu, 2022, p. 15). Scientific research has revealed that fungi often partner and form symbiotic relationships with other species inhabiting our planet. This is evident in the organism’s symbiotic partnership with algae that results in the creation of lichen. This scientific evidence lead the following research question: *can architects and mycelium hybridize in the same way?* This requires establishing a co-creational approach to design that acknowledges multiple autonomies of the architect and the non-human designers. The following technical workflow describes a feedback system developed between the architect’ aesthetic and fungi’s agency of growth. The objective of this developed dialogue is to characterise the attributes of the fungi as a designer and identify their affordances to architectural design and develop methodologies for creating non-indexical formations, capable of hybridising the aesthetics and scale of the fungus with the architect’s design intentions. This developed dialogue and feedback systems between the human and biological agency is described in what follows.

## Mycelium agency

**Fig. 1:**
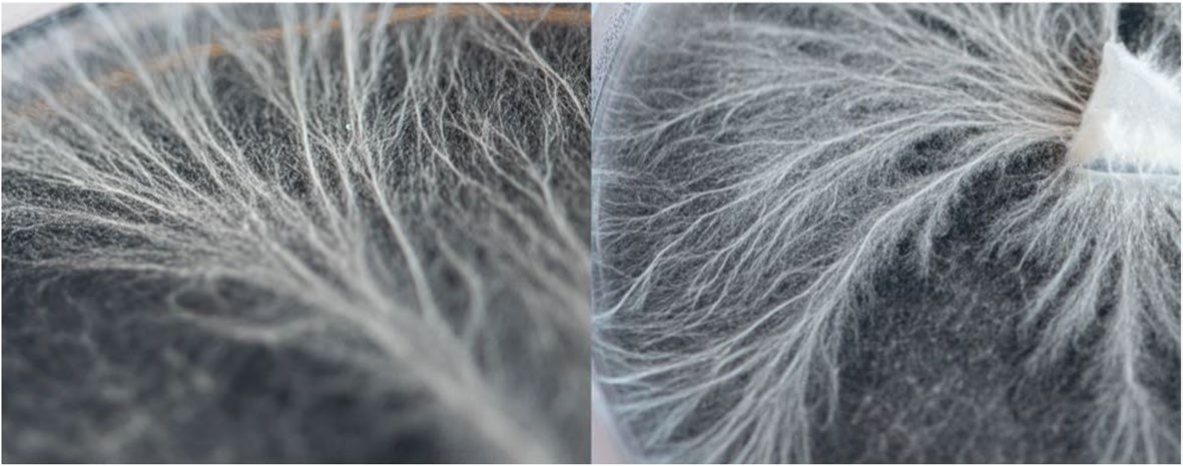
Mycelium growing inside a series of Petri dishes. Demonstrating qualities of rhizomorph entangled patterns of growth, mycelia spreads its hyphae tips in order to explore its surroundings

Methodologically, this project involved the following stages: (i) preparing and applying the mycelium for growth on petri dishes; (ii) scanning the form; (iii) 3D printing the derived form using mycelium extraction, clay and agricultural waste; and (iv) incubating the forms and (vi) continuing the cyclic feedback systems of scanning mycelia data and extruding so that form is generated in real time. These processes involved the introduction of a scanning technology, developed computational algorithms, as well as developing a new mycelium based mixture for 3D printing the forms. In order to work with the organism intricate patterns of growth on a micro-scale, mycelium was initially grown on a series of petri dishes containing agar. Demonstrated in Fig. 1, this medium of growth was selected due to its capability to support a micro-scale growth pattern known as rhizomorph. To cultivate rhizomorph growth, mycelium was grown and sliced into 3 mm x 3 mm pieces. In a sterile environment, one slice of spawn was transplanted into a petri dish containing an agar medium. During the mycelium’s growth, the petri dishes where stored in a dark, temperature controlled, and humid room to encourage cultivation.

**Fig. 2:**
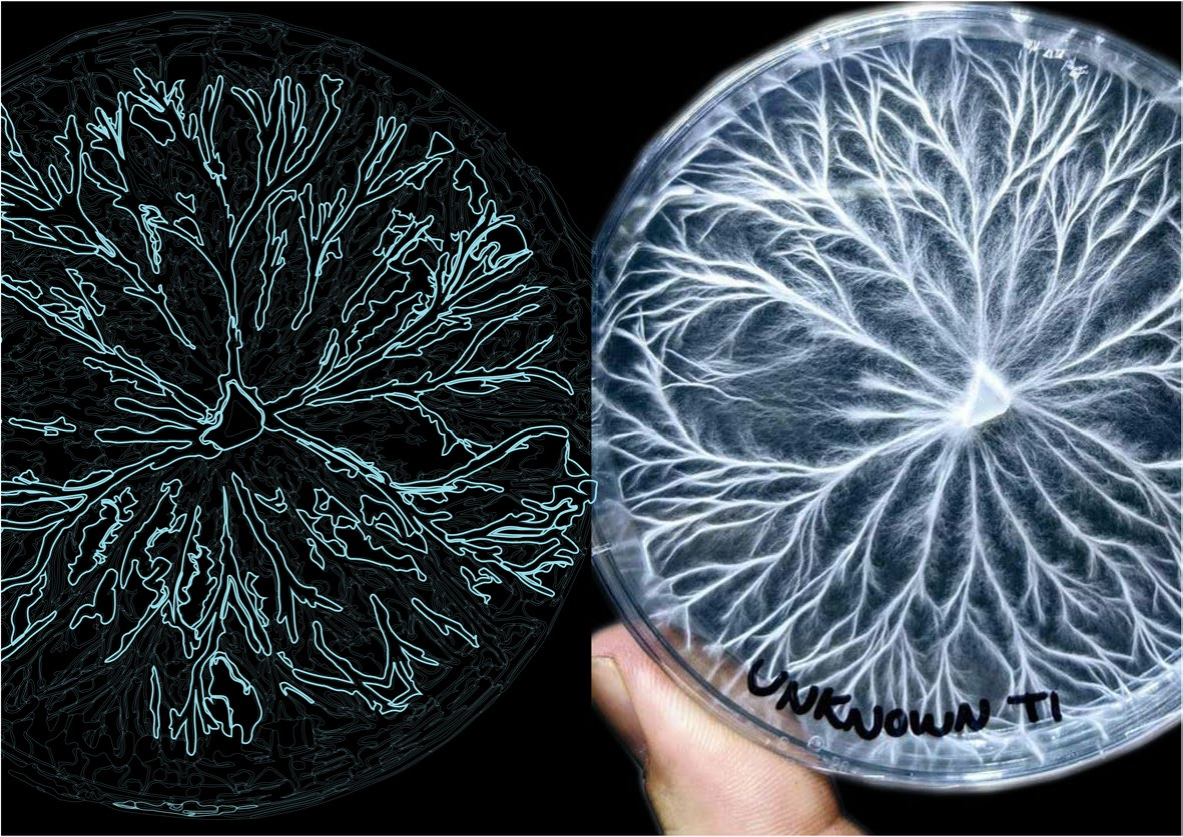
Petri dishes containing mycelia growth (right) and the generated computational scan (left). Image of mycelium growth sourced from unknown online source

In order to convert these intricate patterns of growth from the physical to the digital realm, a digital microscopic web camera with 1080P resolution was utilized in order to track and computationally map the mycelium’s dense fibrous network at the micro scale. Through a developed algorithm based on the logic of edge detection, computational agents traced over mycelia’s patterns of growth, converting the once static image into a complex labyrinths of polylines. This computational logic of boundary edge detection was deployed in order to trace around the organisms intricate patterns of growth, populating both the inner and outer parts of the polylines. This simultaneously generates new pathways from the organisms data and ensured that the fungi’s microscopic detail was not diluted when transferring the physical data into the digital realm. Each living hyphae strand was therefore captured and represented accurately. Using an additional process of color detection, the algorithm was programmed to eliminate unnecessary background interference. This occurred by filtering out forms that did not represent the mycelium’s distinct white flourishing color. This procedure enabled the mycelium’s intricate physical data to be accurately represented in the digital realm. As a result, a series of delicate computational drawings were generated, representing the organisms agency and autonomy of uninhibited growth (demonstrated in Fig. 2). Here computational form, accurately captures characteristics of mycelia’s rhizomorph growth such as: branching, fusing, entanglement, bifurcation and webbing, all which are visible at the micro-scale. Particularly noticeable are the interweaving hyphae tips as they bifurcate, separate and form new connections, resulting in root clusters of entanglement, attracting itself- to itself.

## Architectural design agency

In order to hybridise architectural design intention with the organism’s agency, an additional algorithm based on the logic stigmergic principals was implemented in order to intertwine the volatile nature of mycelium with restrained computational algorithms. The aim of this process was to form a shared inter-species space to which, each creator may contribute, according to their unique affordances. To do so, stigmergic principals were deployed in order to generate self-organizing systems.

Stemming from the Greek words *stigma*, meaning ‘sting’, and *ergon*, meaning ‘work’, stigmergy describes a form of indirect communication in a system of swarm intelligence. The insect world provides fascinating examples for stigmergy. For example, the trail that ants create leading from their nest to a food source is produced through stigmergy (Sumpter & Beekman, 2003). Stigmergy is based on the principle of traces left in the environment by an individual action, stimulate the performance of a succeeding action by the same or different agent (Grass´e, 1959), an external creative ‘design brain’ may be formed, embracing the inputs of the co-creators. This ‘trail’ is strengthened or weakened through a feedback system—when more ants enter the trail, the pheromone signalling strengthens. However, when the food source depletes and ants leave the trail, the pheromone evaporates and the trail dissipates (Meyboom & Reeves, 2013). It is the balance between positive and negative feedback that keeps a system in a dynamic equilibrium, from which self-organised, complex order emerges (Snooks, 2014.)

Since its discovery in entomology, stigmergy has become widely applied in other fields, particularly computational science. An ‘external brain’ of a self-organising system is formed by employing stigmergic algorithms for managing large data sets and coordinating networks of information trafficking. Similar to ants creating a trail by leaving traces of pheromones across their path, in the digital realm, these characteristics of trailing are enabled through computational agents. Within the field of computational architecture, many designers have utilised the principals of stigmergy to generate intricate fibrous geometries. Snooks (2014) for example, utilizes stigmergic principals in the generation of form as it “provides a method of incorporating architectural geometry in the generative process” (p. 191).

**Fig. 3:**
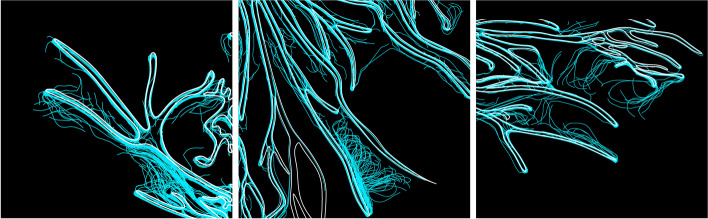
shows the computational agents tracing around the mycelium original polylines (white) and generating entangled systems according to the rules set by the designers within the computational algorithm (blue)


The present research draws upon these ideas and extends them beyond the computational realm by exploring interspecies stigmergic processes in co-creating of hybridised designs. Similar to mycelia growth, here too an ‘external brain’ of a self-organizing system is formed by employing stigmergic algorithms for managing large data sets and coordinating networks of information trafficking. Through self-organizing algorithms that are attracted to predetermined paths, stigmergy is utilized in novel ways that enable architectural design intent to follow the predetermined paths set by the fungus. In analogy to the ant trail, here too the mycelium’s ‘pheromones’ lead the architect along their trails and the architect responds to these trails through a series of developed rules and restraints. Demonstrated in Fig. 3, the pre-determined paths set by the fungus, guide the direction, action and response of the computational agents. This reconceptualizes the notion of architectural intentions, as not pre-fixed, but as continuously evolving in response to changes in stimuli left in the environment by the fungus.

Computational stigmergy consists of four main components: Medium—the method or environment in which the agents communicate with each other; action—rules that direct an agent to interact with the environment or other agents; Trace—a signal left behind by an agent to indirectly communicate with others; and, condition—the events that trigger or allow certain actions to be executed (Navlakha, 2011).

These encoded rules and restraints not only embed my design aesthetic but add a sense of organized complexity to the patterns originated from the fungus. Architectural intention is therefore imbedded into this process by orchestrating the local interactions and micro decisions of computational agents. To do so, the developed algorithm was programed to include the following behavioral protocols: cohesion, separation, flow along curve, seek trail and evaporation of trail. Through the behavior of *cohesion*, the computational agents were instructed to move closely together, generating multiple pathways along the organism original polylines. The *Separation and* behavior caused the caused the agents to remain at a certain distance from the mycelium’s curves, whilst the *Flow along curve* behavior instructed the agents to simultaneously trace over the organism original boundaries whilst generating new labyrinth pathways. *Seek trail,* determined the magnitude in which the computational agents were attracted to the organisms pathways; and *Evaporation of trail*, ensured that the pathways generated by the agents would evaporate over time. This ensured that the aesthetic output of this process remained legible, rather than resulting in intersecting and obscure lines. These behaviors, expose the architectural design intention and determine how computational agents would react to the fungus’s original pathways. Showcased in Fig. 3 simultaneously, there are two contrasting behaviors occurring within this processes. The first being stigmergic behavior were the trails respond to other trails. The second is the computational agent responding the mycelia’s pheromones and trails. Utilizing mycelium’s polylines as the blue print, a stigmergy algorithm was generated in order to seed architectural design intention into hybridized patterns of formation. Through stigmergic computational processes it became possible to embed architectural design intention into forms extracted from nature through self-organizing algorithms that are attracted to predetermined paths. The design intention, was to add further complexity to the mycelia’s original scans in order to generate complex labyrinth systems. The computational agents were therefore programmed to generate new intricate pathways, based on the direction and density of the mycelium’s traces. In order to seed my design intentions within the algorithm, computational behaviors were modified in the following pseudo-code:

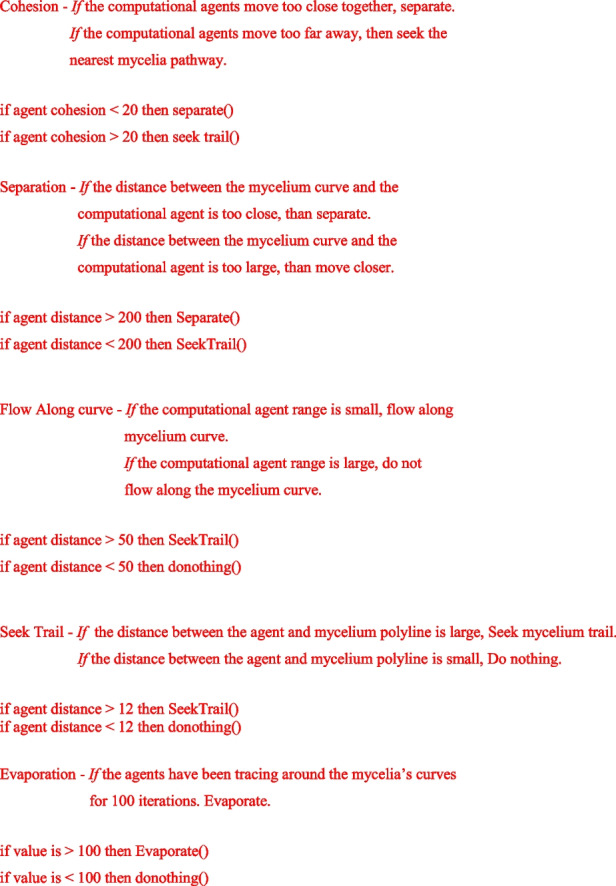


**Fig. 4:**
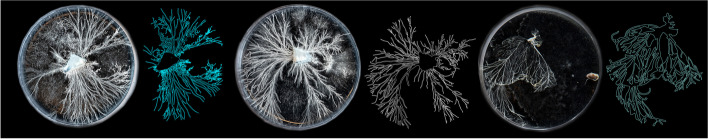
From left to right, image showcasing the organisms original scan, second image showcasing a computational process of color detection, illuminating any unnecessary background noise, third image exposing stigmergic agents trancing around mycelia’s polylines and the final image(right) exposing the hybridized result of the feedback process

**Fig. 5:**
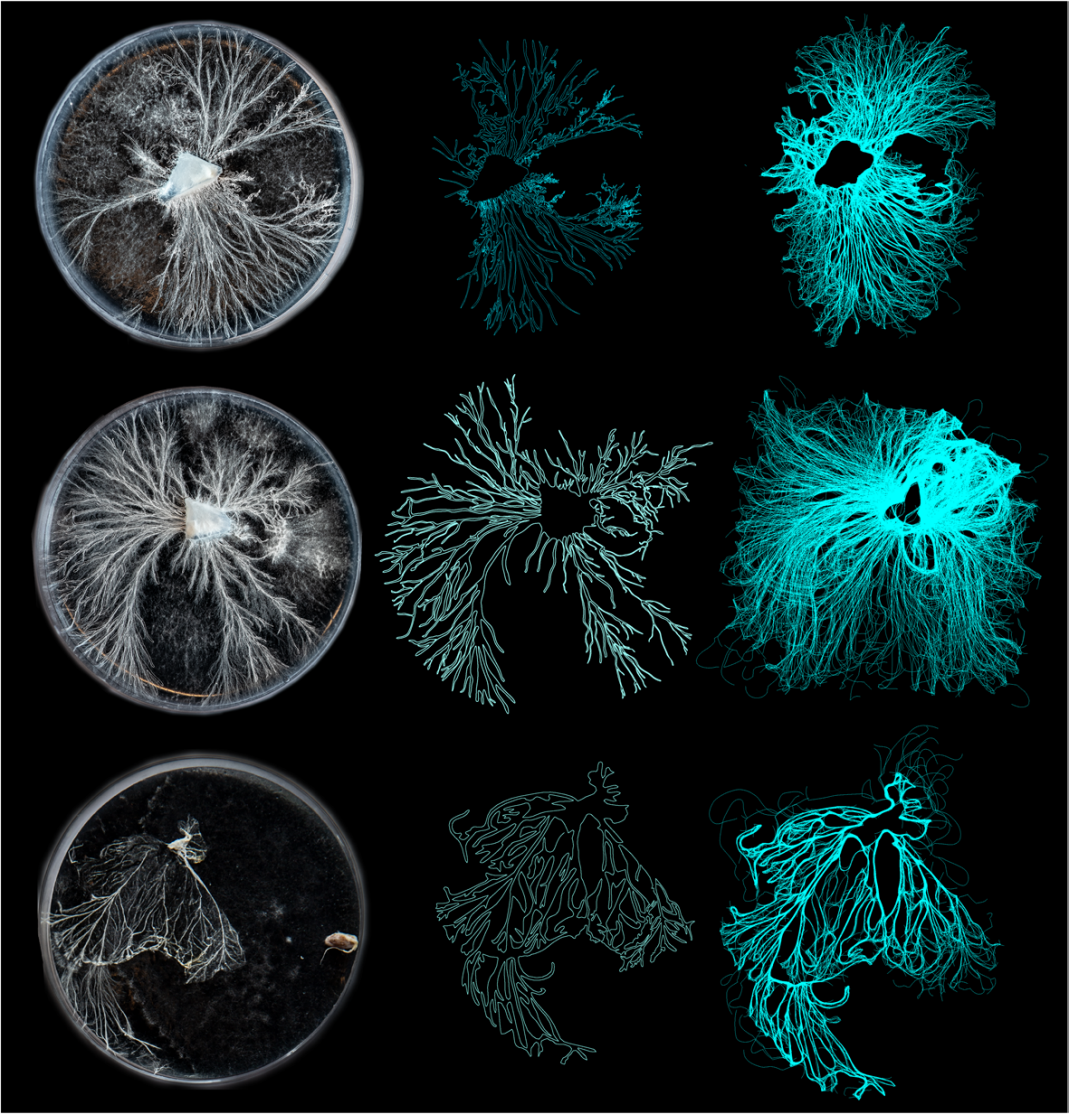
From left to right, Petri dishes containing mycelia growth, computational scan and mycelia drawings and hybridized stigmergic outcome. Computational drawings assisted by Hanying Zhao and Christine O’neill

Responding to the organisms agency, computational agents follow along the designated mycelia trails and simultaneously generate intricate entangled fibrous networks between each hyphae tip. Here, computational agents populate the both the inner and outer boundary edges of the mycelium original polylines. This additional process adds a sense of designed complexity to the mycelium’s web, by generating entangled connections between the hyphae tip. Figure 4 exposes this gradual evolution from the mycelia’s original scan to the stigmergic infusions and finally the hybridized result. The sequential steps of growing the organism within the petri dish, the computational scan and applying the stigmergic algorithm which is seeded with architectural design intention is demonstrated in Fig. 5.

**Fig. 6:**
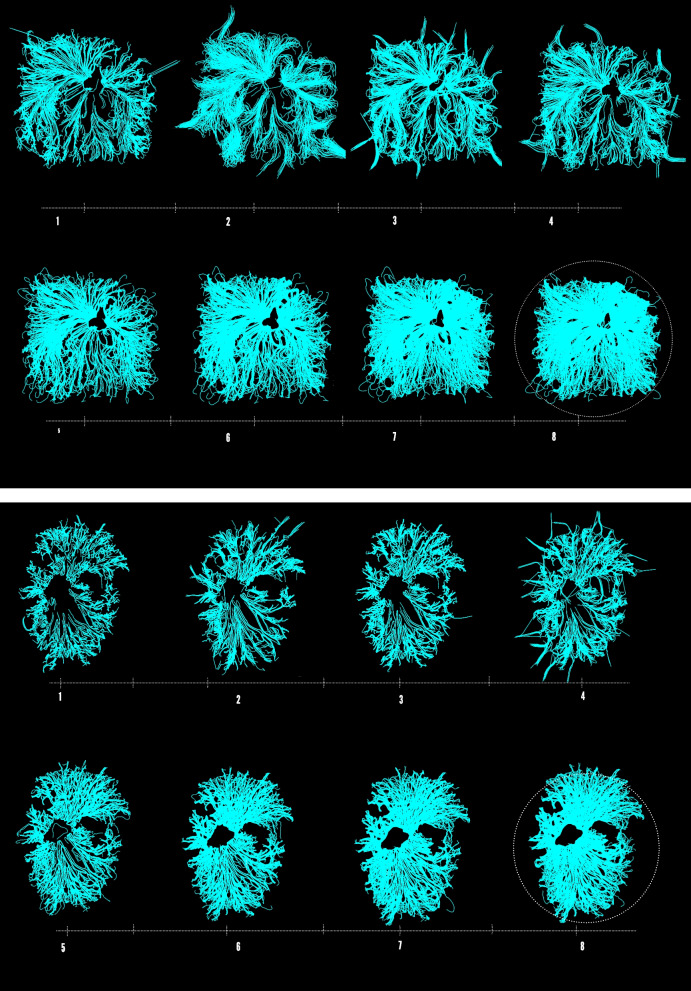
Timeline exposing the gradual entanglement of mycelia’s and architectural agencies. This is initiated from the mycelia’s original scan (on the far left) and hybridized result (on the far right)

**Fig. 7:**
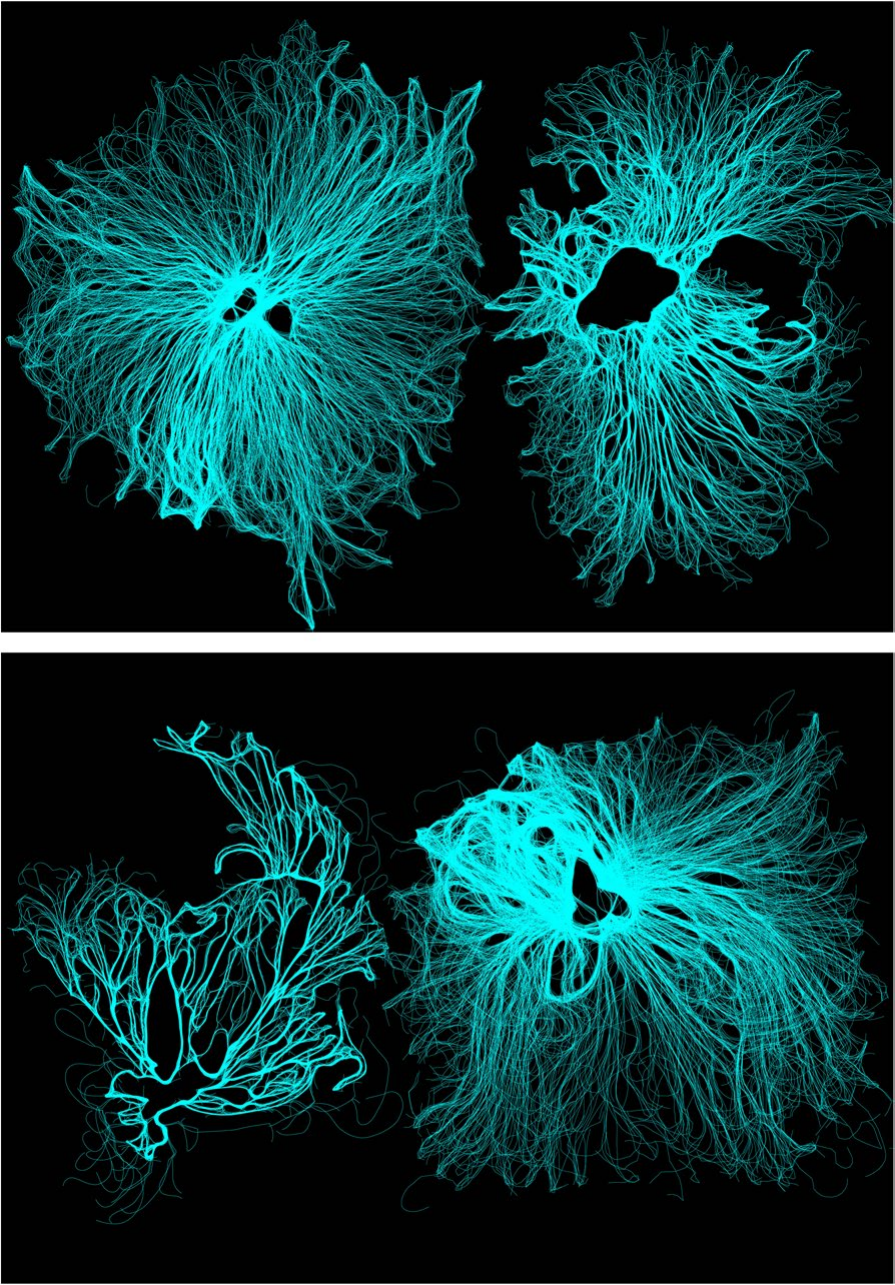
Interspecies Hybridized outcomes of the entanglement between mycelia’s and architectural agencies

In order to determine at which stage of this algorithmic process achieves true hybridity, a timeline was generated, illustrating the gradual entanglement of architectural aesthetic and mycelia agency over time. Demonstrated in Fig. 6, this timeline exposes the formal outcomes, initiating from the moment in which the two agencies first meet to the moment in which the agencies have been engaging with one another over an extended period of time. When the two agencies initially meet, the first iteration of form displays qualities which strongly resemble the mycelium’s original polylines. Although the form is slightly more detailed, true hybridization and novel qualities have yet to emerge. It seems that only when both agencies have intertwined with one another over an extended period of time, fascinating results begin to emerge. These formal qualities and results (demonstrated towards the end of the timeline in Fig. 7) do not resemble the architect’s or fungi’s agency but have rather, generated something new. As a result, form resists authorship as it is non-indexical back to either the architect or the fungus’s agency, generating a new self-organized complex order.

For the purposes of form finding, the goal of this process was to draw out the hybrid emergent characteristics between the natural and artificial realms. The results of this hybridization between mycelia and architectural agencies represent novel outcomes, which are non- indexical back to either mycelia’s scan or computational agency. Whilst mycelia’s original polylines have therefore been morphed, mutated and manipulated into an unrecognizable results, the organisms original features of fibrosity, delicacy and complexity still remain. Demonstrated in Fig. 7, a delicate balance of agency and design authority is achieved as characteristic of mycelia growth and stigmergic processes are still present, but have morphed into novel interspecies results.

## Robotic agency

**Fig. 8:**
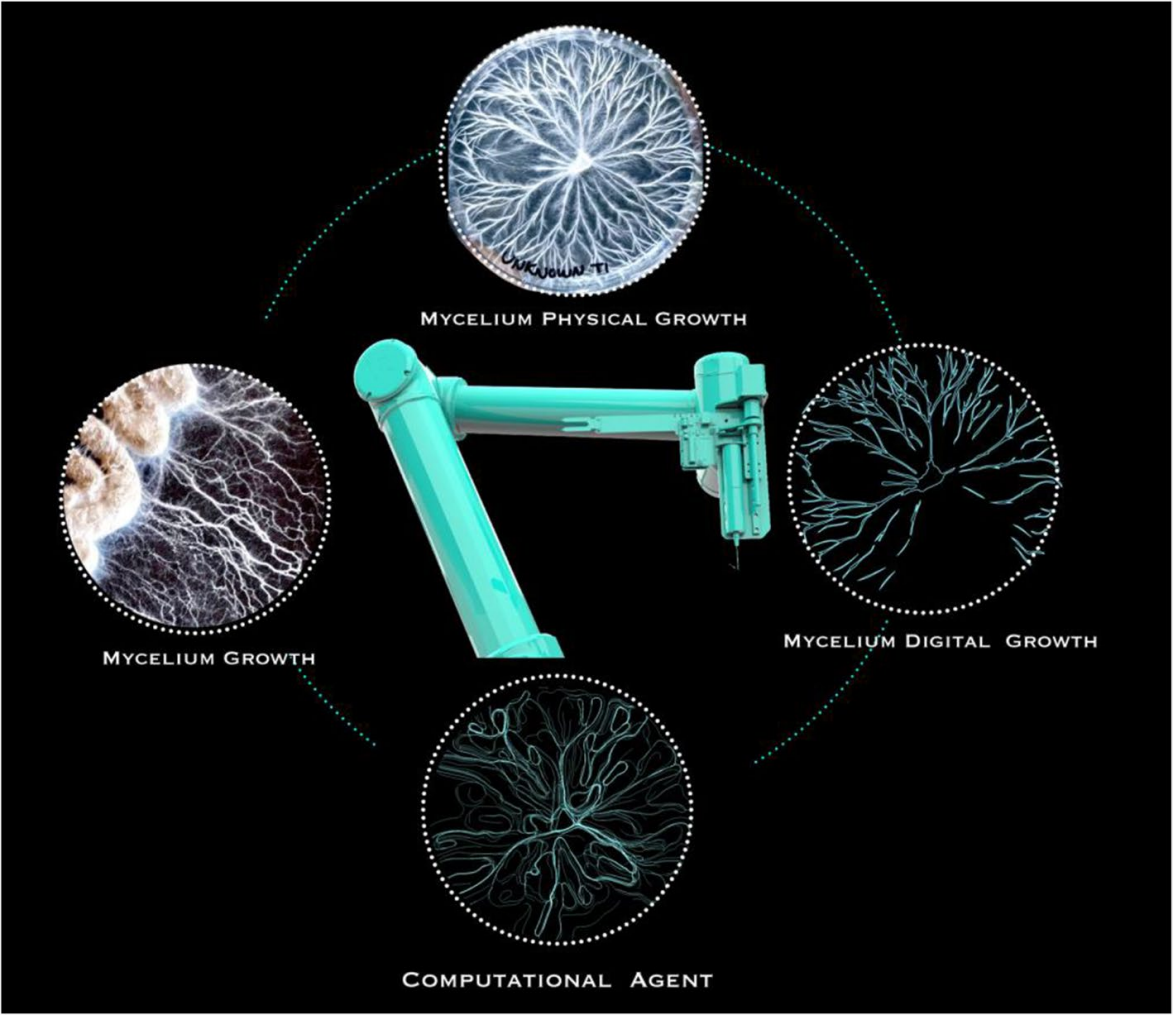
The developed cyclic feedback system between mycelia growth, robotic intervention and computational form. Initiating this feedback system is the growth of mycelia within a series of petri dishes and agar cultures. The organism’s physical patterns of growth are scanned and become inputs for computational form. Stigmergic algorithms are then applied to the mycelia’s polylines, generating hybridized non indexical results. These outcomes are then 3D printed with the biological medium itself. Over time, the robot waits for the organism to grow and responds to fungi growth by initiating the feedback system of scanning and extruding once again

In order to bring these hybridized output forms back into the physical environment and continue this cyclic feedback system between the natural and the artificial, InterspeciesForms where robotically extruded with the mycelium medium itself. In comparison to existing projects that 3D print mycelium, mycelia was kept alive in order to enable its patterns of growth to thrive and contribute to the create of form and robotic movement. Demonstrated in Fig. 8, a feedback system and direct dialogue is developed between biological, architectural and robotic agencies as mycelia growth becomes impute into computational form and robotic movement. This technical process is described in what follows:

A customized mixture of mycelium, clay and agricultural waste was created in order to robotically fabricate the biological medium and encourage growth. This mixture consisted of agricultural waste, which provided nutrients of the organism to thrive and clay which acted as a natural binder for the living fibbers to adhere to. With the outbreak of COVID-19, these robotic tests came to an abrupt stop, as the university was shut down. In the absence of access to an industrial universal robot, I shifted to using Virtual Reality (VR). The HoloLens is an opti cal, head-mounted display (HMD) that composites virtual content with the user’s field of view by rendering to a transparent stereoscopic waveguide display (Jahn, 2019). This application allowed me to visualise the mycelium’s computational digital form in the physical environment. Whilst these geometries were once robotically extruded, an electric corking gun was utilised to trace over the holographic forms, manually extruding the customised mixture of mycelium, clay and agricultural waste. By transferring the robotic workflow from the robotic into the realm of augmented reality, a sense of handcraft-based production began to arise, blurring the analogue and digital approaches for making forms. Once the biological medium was extruded and incubated, the mycelium’s growth began to flourish over a seven-day period, thus continuing this cyclic process of scanning and extruding. Although the extrusions lacked the precision that the robotic could achieve, this temporary feedback system provided key findings, regarding: (i) the ideal mixture of clay, mycelium, waste ratio; and (ii) the observation of mycelium’s reaction once extruded. Once access to robotic facilities resumed, the research experiments involving robotic intervention could continue. In comparison to the extrusions conducted by hand, a higher level of precision was achieved, exposing the design tectonics that were generated from this process of robotic cyclitic feedback systems.

Each hybridized form was 3D printed at the scale of 300 mm x 400 mm. The size of the forms could not identically match the original size of the petri dish derived forms (200 mm diameter), due to limitations set by the nozzle size and the need to prevent clogging. The following steps were taken to test the points mentioned in the creation of the form:

A. Inoculation. Mycelium was firstly inoculated and grown on a mixture of wood chips and paper pulp. Over a seven day period this mixture was incubated with controlled temperature (24–30 °C) and humidity (90%), under a greenhouse tent. This chamber was kept sterile to prevent bacteria growth, while enabling sufficient natural light to pass through.
B. Living filament. Once the mycelium flourished in growth, over a seven-day period, it was introduced to the clay medium. Earthenware clay was utilized due to its porous aerated structure, which enabled the mycelia to seep through and eventually degrade the substrate. Both the clay and mycelium bio composite mixture were fused together, generating a living paste to robotically extrude.
C. Fabrication. Utilizing a customized clay extruder on a Universal Robot (UR), the mycelium mixture was 3D printed according to the following protocol: (i) Nozzle Height: 23.1 mm from Surface; (ii) Nozzle Width: 5 mm; (iii) Print Speed: 10 mm/s; (iv) Flow Rate: 58%; (v) Layer Height:; 1.5 mm; and (vi) Air Pressure: 50 PSI.

**Fig. 9:**
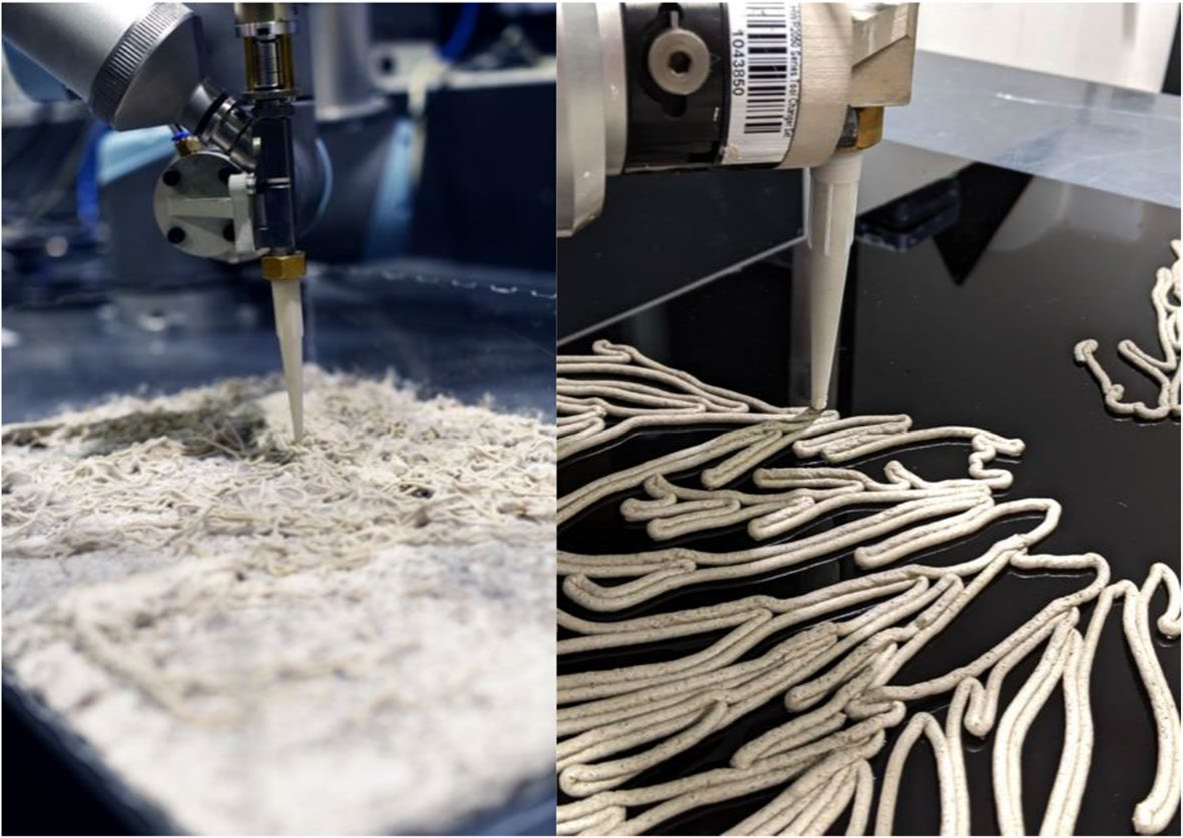
Image showcases the mixture of agricultural waste including mycelium, coffee grinds, paper pulp and hemps seeds being extruded from the Universal-10 robot

Images of the clay-mycelium medium being robotically extruded and growing are shown in Fig. 9.

## Interspecies forms

**Fig. 10:**
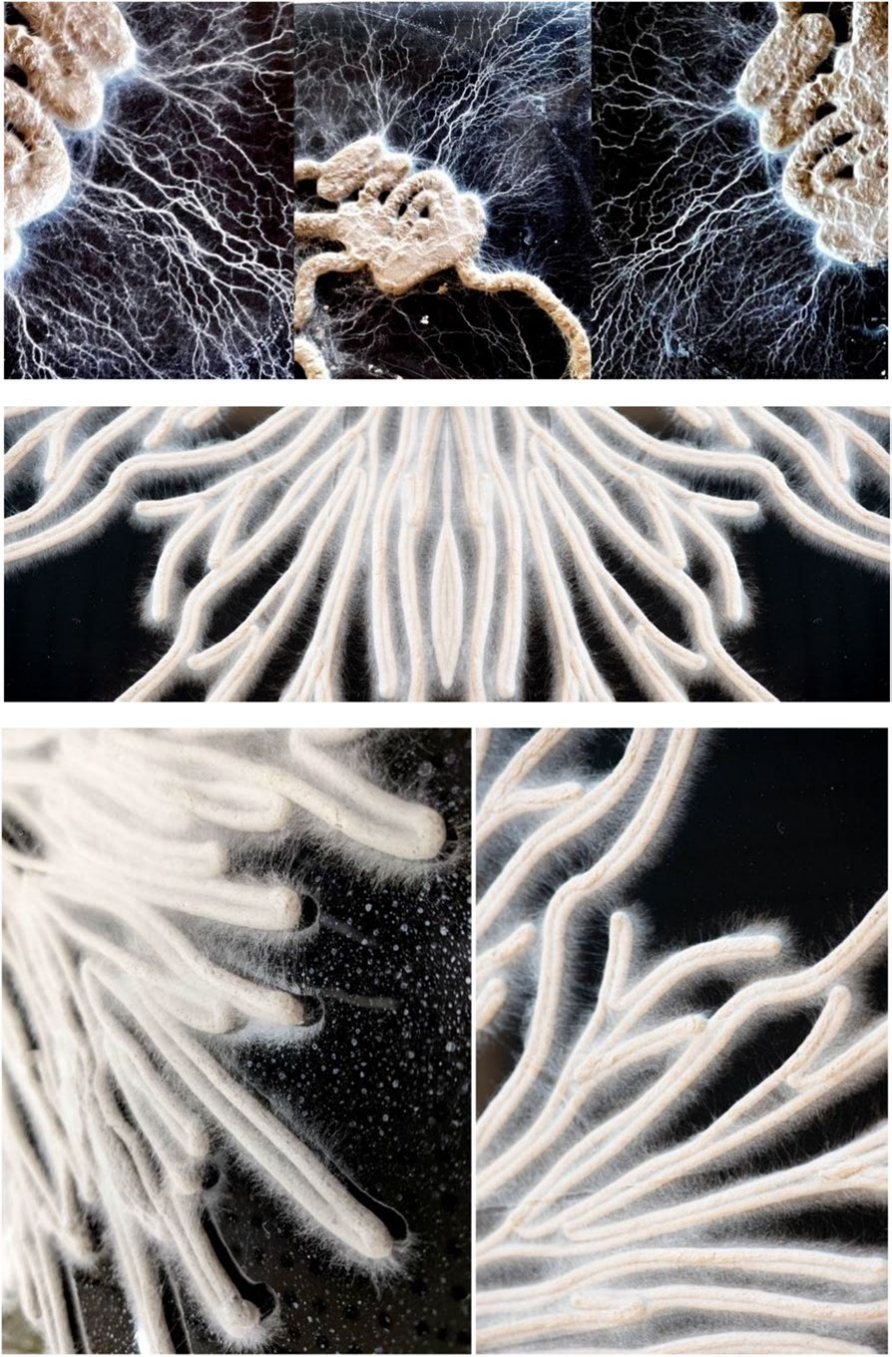
Image showcases the organism grow from the living extrusions by extending its hyphae tips away from its designated path that the robot extruded

Once the organism was 3D printed, the organism began to grow from the living extrusions by extending its hyphae tips away from its designated path that the robot extruded (shown in Fig. 10). This behaviour not only exposed the organism agency but asserted its autonomy, that the living cannot be completely controlled.

**Fig. 11:**
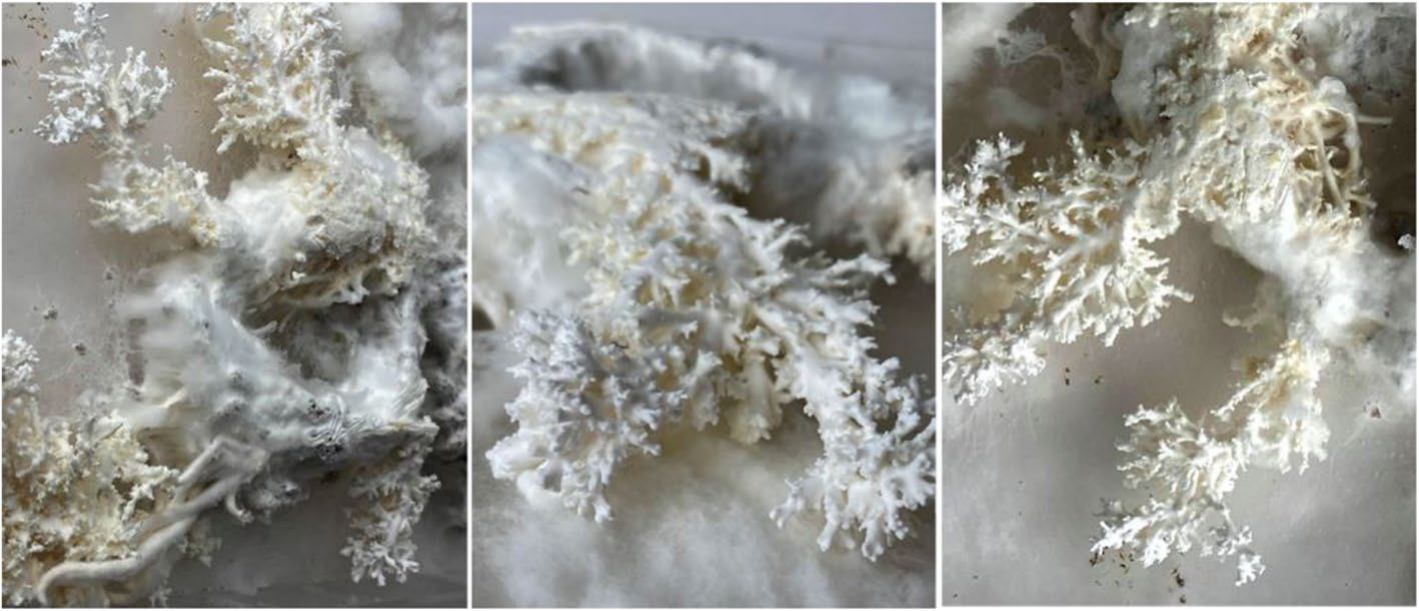
Clay infused with mycelia robotic extrusions. Overtime, the mycelium began to grow from the organic mixture and wondered around the surface area in search for additional nutrients

During this period, three types of growth behaviour were observed. The first was noted by change of texture and color. Whilst initially the designed form had a smooth grey exterior – resembling the appearance of clay, over time the extrusions turned hairy, fluffy and furry, resembling the texture of hyphae, imbedded in the extrusion. Shown in Fig. 11, a second noted change was that the form began to fruit. Long cylindrical mushrooms began to blossom and tower over the tapestry. Finally, it was observed that over the course of seven days, the mycelium increasingly biodegraded the clay mixture substrate. When doing so, it began wondering around its surrounding in search for additional nutrients to absorb. This was fascinating to observe, as the fungus was no longer constrained by the extruded form from which it originated, but rather began to affirm its own agency of growth. A distinct set of generative patterns of growth and characteristics such as branching and fusing began to emerge. This volatile behaviour resulted in vein like formations, not set by the architect. Here the fungus clearly asserted its autonomy over the design.

**Fig. 12:**
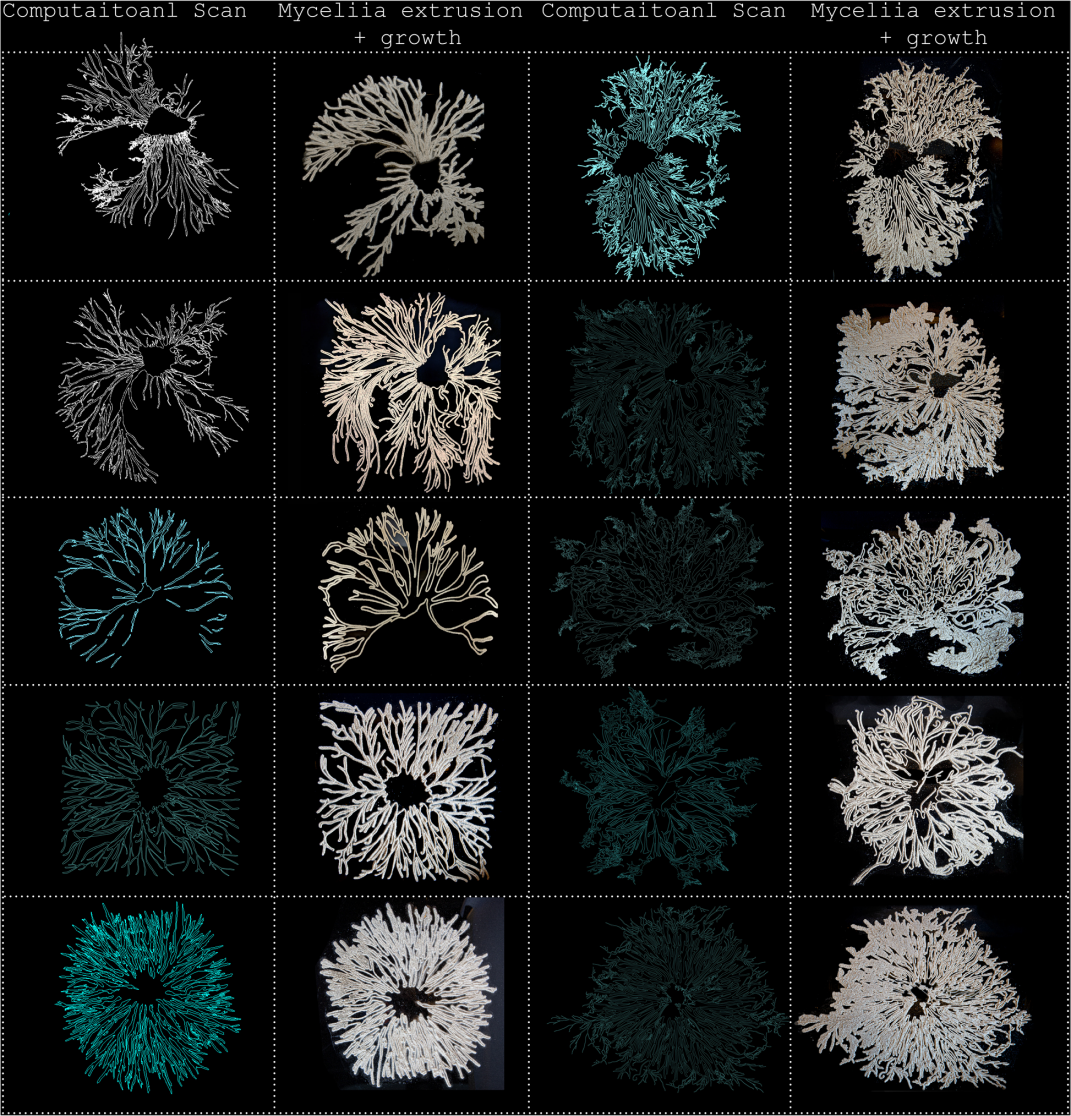
Exposes a catalogue of forms that began from the mycelium original computational scan (left), the architect infusing his or hers design aesthetic through stigmergic principals and printing the living form. Once the organism begins to flourish and grow beyond its set boundaries, an additional scan is conducted and manipulated through stigmergic based algorithm. This new mutated form is once again 3D printed (right), continuing this cyclic feedback systems between the architect and fungi’s agency of growth

Once the organism began to divert and grow beyond its set boundaries, an additional scan was conducted in order to repeat the cyclitic feedback prosses of growing- scanning and extruding. Resulting from this feedback systems, a catalogue of forms shown in Fig. 12 were generated exposing emergent qualities of this developed feedback systems between the natural and artificial realms. Whilst fungi generates the first ‘move’ be exposing its flourishing, inhibited patterns of growth, the architect designs with these patterns of growth by contributing their own design aesthetic. Once this hybridized form has been printed and grown, the developed feedback systems continues as form mutates and evolves over time.

## Results

Through this developed feedback system the formation of architecture is directly driven by mycelium behaviour, rather than an a priori parametric model or generative algorithm. Methodologically, the contributions of InterspeciesForms include the scanning mycelia growth in order to computationally visualize its patterns of growth, utilizing mycelia growth as impute to developed algorithms based on stigmergic behaviours, generating hybridized outcomes from the co-creation of architectural and mycelia agencies, and developing behavioural based feedback systems. The findings of this research conclude that by applying stigmergy into biodesign, new opportunities for co-creation and a hybridized novel design language may arise. By embracing non-human aesthetics within architectural design, multi-species and biocentric forms may arise. Furthermore, it appears that the allocation of high levels of design autonomy to the fungus, has led to the creation of a new set of forms that embrace the strange and highly volatile nature of the mycelium. No two from outputs in this process were the same, where each fabricated design presented a contrasting set of biological patterns of growth. Stigmergy therefore provided a path for co-designing with a non-human organism by which mycelium may thrive uninterruptedly while contributing to the creation of hybridized- Interspecies forms.

The contribution of this research to the field of Architecture is providing a new definition to the concept ‘hybridization’ within design. Methodologically, the research presents new methods for collaboration with organisms, and conceptually it offers a strange and novel set of formal tectonics to arise, contributing to architectural design discipline. Furthermore, the findings provide the empirical grounding for reconceptualising and redefining the notion of *hybridisation* in architectural design. The study further suggests that new biologically driven design processes may emerge by architects embracing nature’s intelligence and behavioural characteristics. The importance of generating true hybridisation between architectural design intention and biological agencies enables a dynamic shift from stable into highly volatile form finding methodologies. In order for novel, interspecies formal outcomes to emerge, the architect is required to reconceptualise design autonomy and control in the generation of form. Resisting sole authorship, these highly volatile systems, which I refer to as *hybridised formations* generate novel relationships and feedbacks, rather than controlled or predictable outcomes. Potent with architectural possibilities, *InterspeciesForms* therefore embraces the highly volatile and seeks the unknown, in order to generate a new interspecies architectural language between human and non-human entities. The purpose of this research is ultimately to be utilized as a template for other designer to expand their imaginations and evolve preconceived notions of architectural form. ‘InterspeciesForms’ therefore explores the possibilities of organisms that transcend its application of a ‘sustainable’ building material within the field of architecture and advancing the field of bio design. This research therefore demonstrates a novel- hybrid design language, that embraces the aesthetics of non-human organisms, giving rise to non-indexical forms for architectural design purposes. This research demonstrates novel design methods for co-creating with non-human organisms that may give rise to new non-indexical formations for architectural design purposes. Through this developed feedback system the formation of architecture is directly driven by mycelium behaviour, rather than an a priori parametric model or generative algorithm.

Reflecting upon the limitations of this research, the hybridized results remained on the two-dimensional scale and did not eventuate into three-dimensional forms. Due to a lack of access to high resolution vision systems that could capture the organism growth in the 3D realm, the scanning of mycelia’s was limited to the two-dimensional scale. Thus the stigmergic response mirrored this scale and generated two dimensional hybridized outcomes. In order to advance this established feedback systems further, higher resolution vison systems are required to capture the mycelia’s growth within the petri dish on the three-dimensional scale. Rather than responding in the two-dimensional realm, the architect may generate 3D forms which emerge from the interaction of architectural aesthetic and the behavior of mycelia growth.

## Data Availability

Data sharing is not applicable to this article as no datasets were generated or analyzed during the current study.

## Footnotes

1 The term flourishing is utilized throughout this research appear when referring to the uninhibited growth of mycelium.

## References

[CR1] Alima Natalie (2022). Interspecies Forms.

[CR2] Darwin C (1859). On the origin of species: Natural selection and evolution in biology.

[CR3] Grass é Pier Paul (1959). The automatic regulations of collective behavior of social insect and stigmergy. Journal de psychologie normale et pathologique (Paris).

[CR4] Haneef M, Ceseracciu L, Canale C, Bayer IS, Heredia-Guerrero JA, Athanassiou A (2017). Advanced Materials From Fungal Mycelium: Fabrication and Tuning of Physical Properties. Scientific Reports.

[CR5] Heisel F, Lee J, Schlesier K, Rippmann M, Saeidi N, Javadian A, Nugroho AR, Mele TV, Block P, Hebel DE (2017). Design, Cultivation and Application of Load-Bearing Mycelium Components The MycoTree at the 2017 Seoul Biennale of Architecture and Urbanism. International Journal of Sustainable Energy Development.

[CR6] Holt GA, Mcintyre G, Flagg D, Bayer E, Wanjura JD, Pelletier MG (2012). Fungal Mycelium and Cotton Plant Materials in the Manufacture of Biodegradable Molded Packaging Material: Evaluation Study of Select Blends of Cotton Byproducts. Journal of Biobased Materials and Bioenergy.

[CR7] Jones M, Mautner A, Luenco S, Bismarck A, John S (2018). Waste derived low cost mycelium composite construction materials with improved fire saftey. Fire and Safety Materials.

[CR8] Jones M, Mautner A, Luenco S, Bismarck A, John S (2020). Engineered mycelium composite construction materials from fungal biorefineries: A critical review. Materials & Design.

[CR9] Lim M, Shu Y (2022). The Future is Fungi : How Fungi Can Feed Us, Heal Us, Free Us and Save Our World.

[CR10] Meyboom Annalisa, Reeves David (2013). Stigmergic Space. Conference paper, ACADIA, Adaptive Architecture, Cambridge, Canada.

[CR11] Navlakha S, Bar-Joseph Z (2011). Algorithms in nature: The convergence of systems biology and computational thinking. Molecular Systems Biology.

[CR12] Sheldrake M (2021). ENTANGLED LIFE : How fungi make our worlds, change our minds & shape our futures.

[CR13] Simard SW, Perry DA, Jones MD, Myrold DD, Durall DM, Molina R (1997). Net transfer of carbon between ectomycorrhizal tree species in the field. Nature.

[CR14] Snooks Roland (2014). Behavioural Formation: Multi-Agent Algorithmic Design Strategies.

[CR15] Sumpter DJ, Beekman M (2003). From nonlinearity to optimality: Pheromone trail foraging by ants. Animal Behaviour.

